# Proteomic profiling of concurrently isolated primary microvascular endothelial cells, pericytes, and vascular smooth muscle cells from adult mouse heart

**DOI:** 10.1038/s41598-022-12749-6

**Published:** 2022-05-25

**Authors:** Zhiping Cao, Jessica Minnier, Lijuan Liu, Kristin L. Lyon Scott, Ashok P. Reddy, Phillip A. Wilmarth, Larry L. David, Anthony P. Barnes, Marjorie R. Grafe, Sanjiv Kaul, Nabil J. Alkayed, Catherine M. Davis

**Affiliations:** 1 The Knight Cardiovascular Institute, Oregon Health and Science University, Portland, OR 97239 USA; 2 Department of Anesthesiology and Perioperative Medicine, Oregon Health and Science University, 3181 SW Sam Jackson Park Road, Portland, OR 97239 USA; 3 School of Public Health, Oregon Health and Science University, Portland, OR 97239 USA; 4 Proteomics Shared Resource, Oregon Health and Science University, Portland, OR 97239 USA

**Keywords:** Proteome informatics, Cardiovascular biology

## Abstract

The microcirculation serves crucial functions in adult heart, distinct from those carried out by epicardial vessels. Microvessels are governed by unique regulatory mechanisms, impairment of which leads to microvessel-specific pathology. There are few treatment options for patients with microvascular heart disease, primarily due to limited understanding of underlying pathology. High throughput mRNA sequencing and protein expression profiling in specific cells can improve our understanding of microvessel biology and disease at the molecular level. Understanding responses of individual microvascular cells to the same physiological or pathophysiological stimuli requires the ability to isolate the specific cell types that comprise the functional units of the microcirculation in the heart, preferably from the same heart, to ensure that different cells have been exposed to the same in-vivo conditions. We developed an integrated process for simultaneous isolation and culture of the main cell types comprising the microcirculation in adult mouse heart: endothelial cells, pericytes, and vascular smooth muscle cells. These cell types were characterized with isobaric labeling quantitative proteomics and mRNA sequencing. We defined microvascular cell proteomes, identified novel protein markers, and confirmed established cell-specific markers. Our results allow identification of unique markers and regulatory proteins that govern microvascular physiology and pathology.

## Introduction

Scientific studies of the pathophysiology of coronary artery disease (CAD) have led to the development of new therapies and to a significant decrease in CAD-related mortality. Up to 50% of patients with angina, who undergo elective coronary angiography are found to have non-obstructive coronary artery disease, in part due to coronary microvascular disease (CMD)^1^. This trend coincides with a shift in cardiovascular disease presentation, where acute myocardial infarctions (MI) due to atherothrombotic plaque rupture are decreasing and the rates of hospitalizations due secondary MI and heart failure are rising. There is also a shift in cardiovascular risk factors with rising rates of obesity, glucose intolerance, and older age^2^. CMD can exist alone or co-exist with CAD, lead to or exacerbate existing ischemic heart disease, and is associated with a significant risk of major adverse cardiovascular events. Unfortunately, our current understanding of CMD is limited, and there are currently no specific therapies to address diseases of small vessels in the heart.

Reducing cardiovascular disease-associated mortality will require better understanding of microvascular function and disease. Coronary microvessels perform key functions supported by unique properties and regulatory mechanisms. Impairment of these mechanisms can lead to CMD. The microcirculation is the site of most vascular resistance and the location of blood-tissue exchange, lymphatic drainage, and immune cell tissue infiltration. These properties allow microvessels, which include arterioles, capillaries, and venules, to regulate myocardial blood flow to match metabolic demands and facilitate immune surveillance of capillary fluid and gas exchange.

The main cell types in microvessels (defined as < 200 µm vessels) are endothelial cells (ECs), pericytes (PCs), and vascular smooth muscle cells (VSMCs). Although the same cells comprise large vessels, there are significant phenotypic and gene expression differences between small and large vessels within the same organ. These differences are not surprising, as they reflect the mechanical, chemical and humoral influences that different size vessels are exposed to. Furthermore, differences in vascular cells have also been reported among different tissues (e.g. heart, brain, or kidney), along the arteriovenous axis (e.g. arteries, capillaries, or veins), over time (vessels from young or old subjects), and with different disease states (e.g. diabetes or hypertension)^3–8^. This remarkable heterogeneity reflects the dynamic nature of blood vessels and their plasticity and adaptability to changes in the surrounding environment. However, this temporal heterogeneity raises a challenge when studying vascular cells separately, as different cells are often isolated form separate animals that have been exposed to different environmental influences. Furthermore, investigators often use different protocols to isolate different cells, which may also influence gene expression in these cells.

Advances in high-throughput analyses of mRNA, protein, and metabolites from individual cells, including single-cell and integrated multi-omic approaches^9,10^, show promise for elucidating the complexity of the interactions among different vascular cells under physiological and pathological conditions. To that end, we have developed a protocol for simultaneous isolation, culture, and mass spectrometry (MS)-based proteomic analysis of the three microvascular cell types from mouse heart. The protocol allows for analysis and co-culture of vascular cells that have been exposed to the same in vivo conditions. Using this approach, we have characterized the proteomes of microvascular ECs, PCs and VSMCs, and have identified novel protein markers of cell type, in addition to confirming established cell-specific markers.

## Results

**Figure 1:**
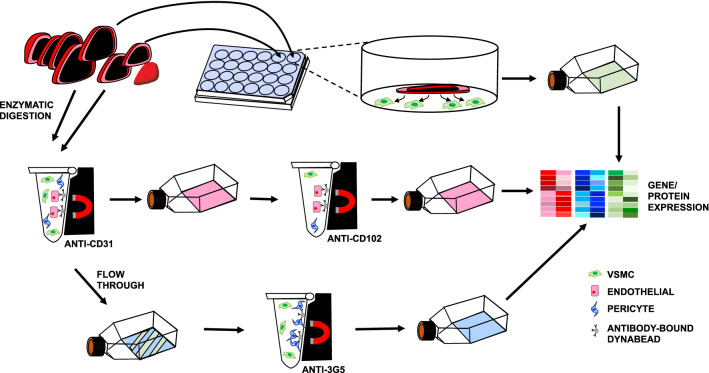
Heart EC, PC, and VSMC isolation protocol. Schematic representation of the isolation and culture process used to generate EC, PC, and VSMC concurrently from mouse hearts. Hearts are isolated and 4 apical slices are taken from each heart to generate SMCs by explant.The remaining heart tissue is digested to generate a single cell suspension. EC cultures are generated by magnetically selecting cells with CD31-conjugated Dynabeads, culturing until confluent and then again magnetically selecting using CD102-conjugated Dynabeads. PCs are isolated by culturing CD31-negative cells until confluent and then selecting using 3G5 ganglioside-conjugated antibody.

**Figure 2:**
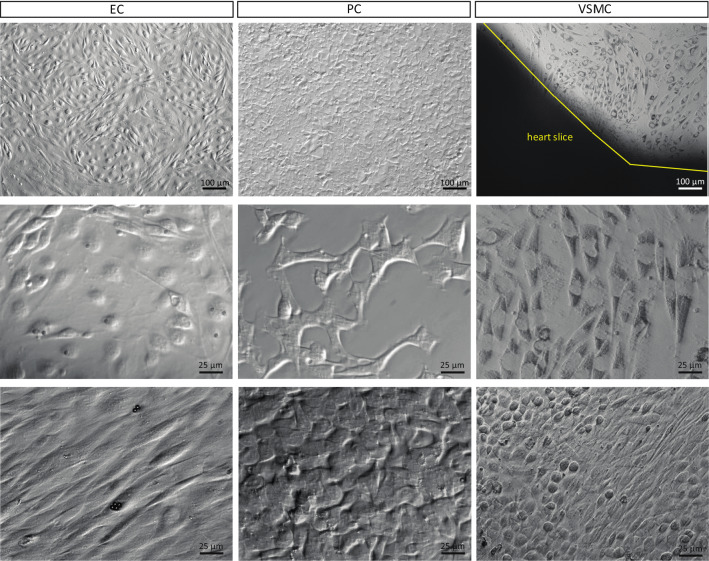
Morphology of cultured ECs, PCs, and VSMCs. Top row: low magnification phase-contrast images of cells, note the typical cobble-stone appearance of the confluent EC culture. Scale bar, 100 µm. Middle row: sub-confluent cultures, with pericytes displaying their characteristic polygonal shape, scale bar 25 µm. Bottom row: high magnification images of confluent cultures; ECs form a uniform monolayer (Dynabeads still present on some cells), PCs maintain their polygonal shape, and VSMCs generate a culture of mixed round and spindle-shape morphology, scale bar 25 µm.

**Figure 3:**
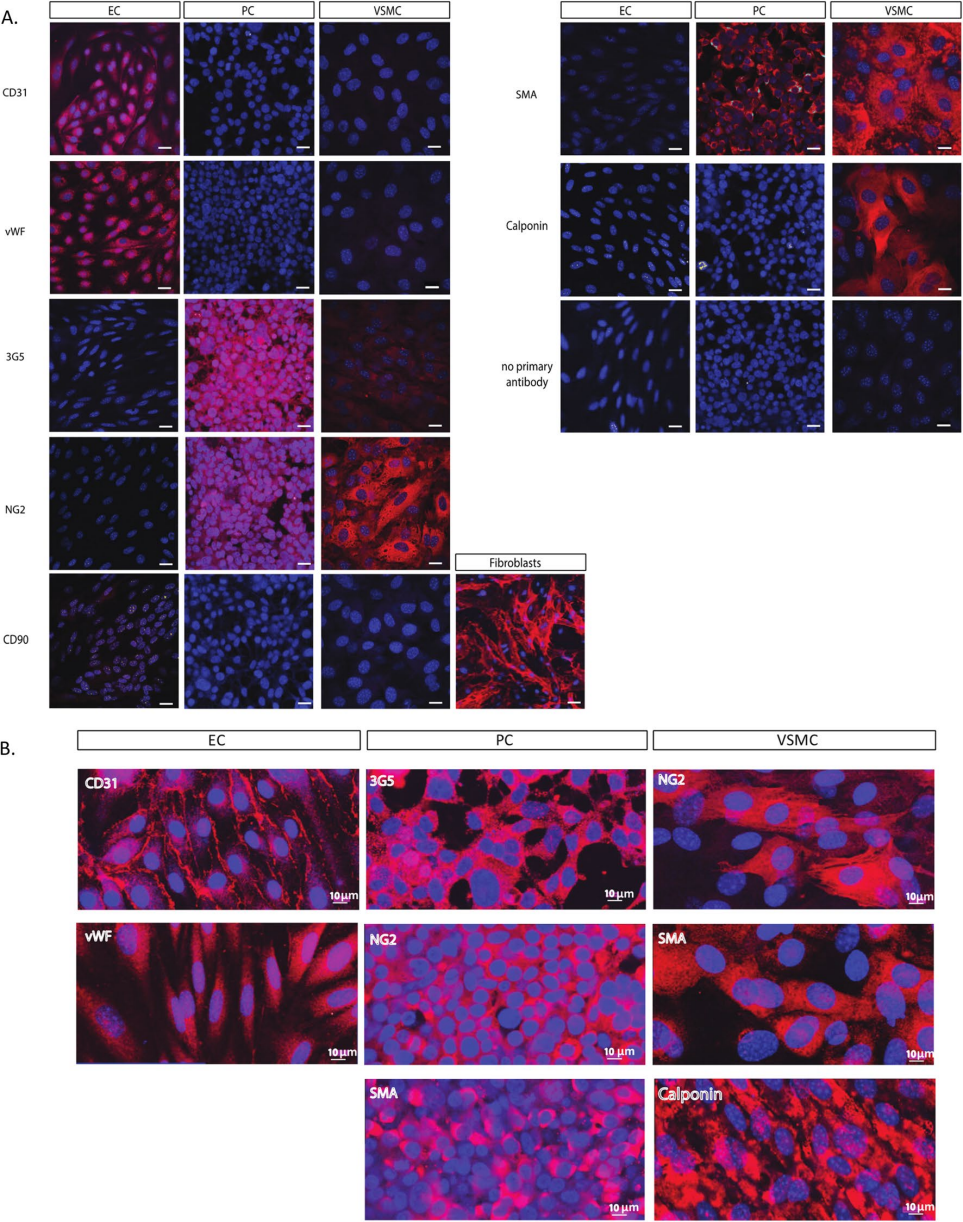
Immunocytochemical validation of heart ECs, PCs, and VSMCs. (**A**) Immunolabeling of ECs (first column), PCs (middle column) and VSMCs (last column) with antibodies against typical cell markers: CD31, PDGFR-β, vWF, 3G5 ganglioside, NG2, SMA and calponin, all in red. Cells are counterstained with Hoechst 33342 (blue). Cells were also immunolabeled with anti-CD90, present in fibroblasts, but not in EC, PC, or VSMC cultures. Scale bar, 20 µm. (**B**) High magnification of cell-specific markers, scale bar, 10 µm.

We developed a workflow for simultaneous isolation and characterization of mouse heart microvascular cells. Figure 1 is a schematic illustration of the workflow used to isolate cells. After isolation, cells are maintained in culture. The phase-contrast images in Fig. 2 demonstrate the typical morphology of the different cell types: cobble-stone appearance of confluent ECs, polygonal PCs, and “peak-valley” VSMC growth pattern^11^ with spindle-shaped (contractile) and round cells (synthetic/proliferative). We further confirmed cell identities using immunocytochemistry with a panel of antibodies against cell-specific proteins. Figure 3 shows positive immunolabeling of ECs, but not PCs or VSMCs, for endothelial markers CD31 (PECAM) and vWF. There was positive labeling of PCs (but not ECs or VSMCs) for pericyte marker 3G5, positive labeling of VSMCs (but not ECs or PCs) for VSMC marker calponin, and positive immunolabeling of VSMCs and PCs (but not ECs) for mural cell markers NG2 and α-SMA. As a negative control, we used the fibroblast marker CD90, which was absent in all three cell types.

**Figure 4:**
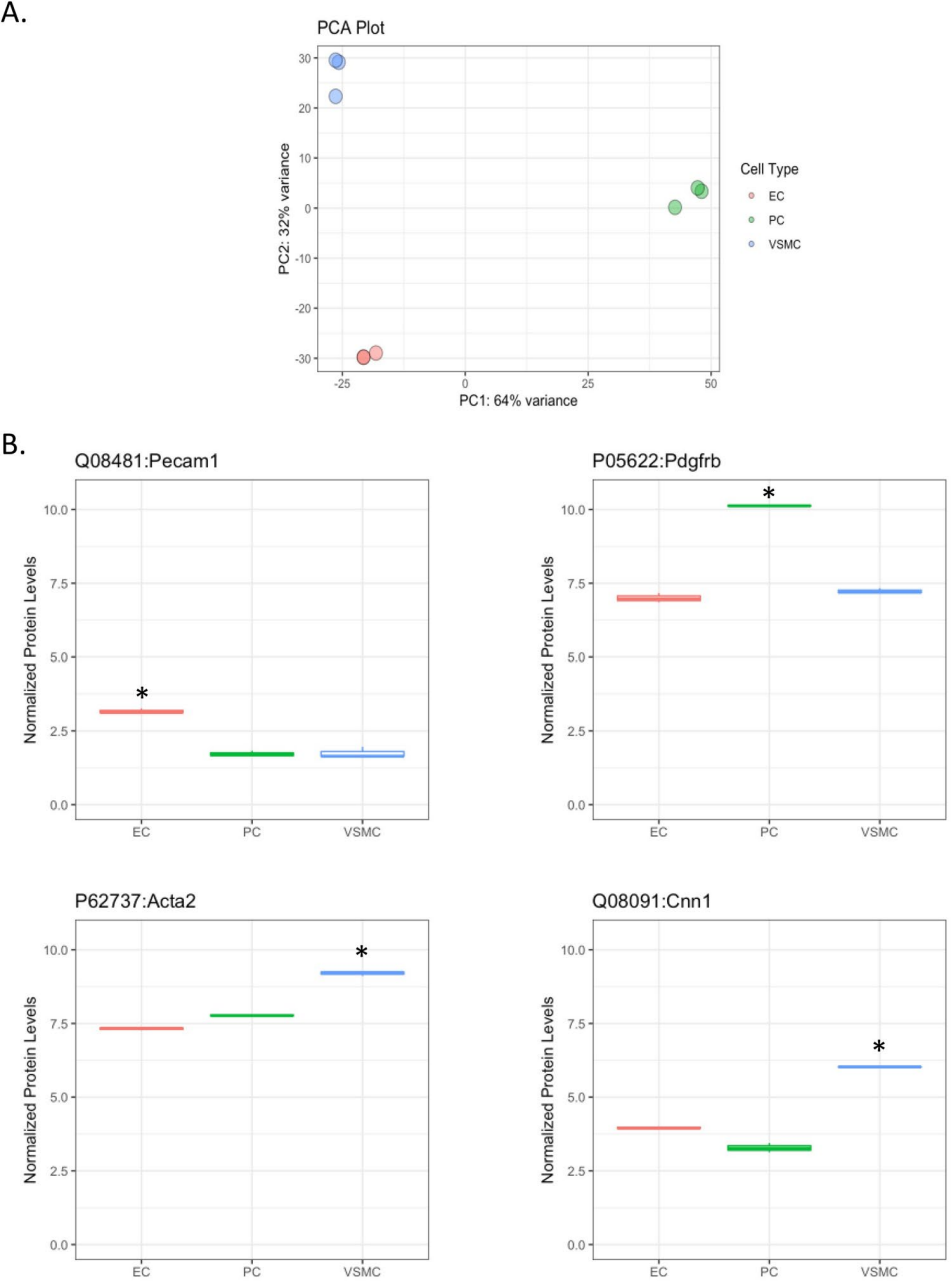
Proteomic analysis shows three distinct cell populations. (**A**) PCA plot showing three distinct and reproducible cell populations upon global proteomic analysis of cultured ECs, PCs, and VSMCs, n = 3. (**B**) Boxplots (median, interquartile range), of proteomic analysis of cell-specific markers. Values were filtered based on FDR rate < 0.05 and then log fold change values compared to the other cell types, n = 3, *p < 0.05, compared to both other cell types (R software package edgeR).

Having established the cell isolation protocol and confirmed cell identities by immunocytochemistry (ICC), we then analyzed each cell type using tandem mass tag (TMT) quantitative proteomics. Figure 4A is principal component analysis (PCA) visualization, showing clear separation between the three cell types, with 3 independent samples representing each cell type overlapping or located very closely to each other. Figure 4B shows levels of cell-specific proteins in the different cell types, further confirming cell identities and validating our cell isolation protocol. Specifically, our analysis shows that levels of the endothelial marker CD31 (PECAM1) is higher in ECs than in other cells, the pericyte marker PDGFRβ was higher in PCs than other cells types, and α-SMA (ACTA2) and calponin (CNN1) were higher in VSMCs compared to the other cells. We also compiled the top 50 most abundant proteins and transcripts, illustrate overlap and carried out Gene Ontology (GO) term analysis, for each cell type (Supplemental Table S1, Supplemental Figs. S1 and S2) after performing bulk RNA sequencing of each cell type using the same isolation and culture methods. The RNA sequencing data have been deposited to GEO with the series number GSE190809. [The data is available for reviewers with token gnefuwiafzwnvcz].

**Figure 5:**
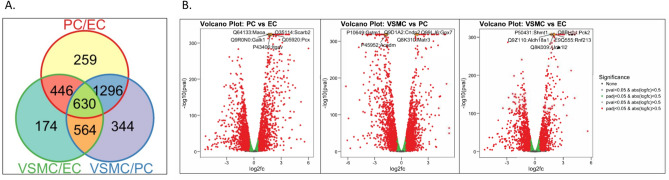
Differential protein profiles of cultured ECs, PCs, and VSMCs. (**A**) VENN diagram depicting number of proteins unique to, and shared between, the 3 cell types. (**B**) Volcano plots showing differential protein expression between cell types, plotting statistical significance (-log10 p-values) versus magnitude of change (Log_2_FC), n = 3.

Overall, 4694 proteins were detected in all three cell types (Supplemental Table S2). Setting a threshold for differentially expressed (DE) proteins at abs(logFC) > 0.5 and hypothesis test FDR < 0.05, pairwise comparisons identified 2834 DE proteins between VSMCs and PCs, 2631 between PCs and ECs, and 1814 between VSMCs and ECs (Fig. 5A). These differences can be visualized in the volcano plots in Fig. 5B, which display statistical significance (*− *log_10_ p-values; y-axis) versus magnitude of change (Log_2_FC). Five proteins with highest fold change and statistical significance are highlighted in orange and labelled in each comparison. The top DE proteins between ECs and PCs were: Maoa, Galk1, Itgav, Scarb2, and Pcx. The top DE proteins between VSMCs and PCs were: Gstm1, Acadm, Cndp2, Gpx7, and Matr3. The top DE proteins between VSMCs and ECs were: Shmt1, Aldh18a1, Aldh1l2, Pck2, and Rnf213.

**Figure 6:**
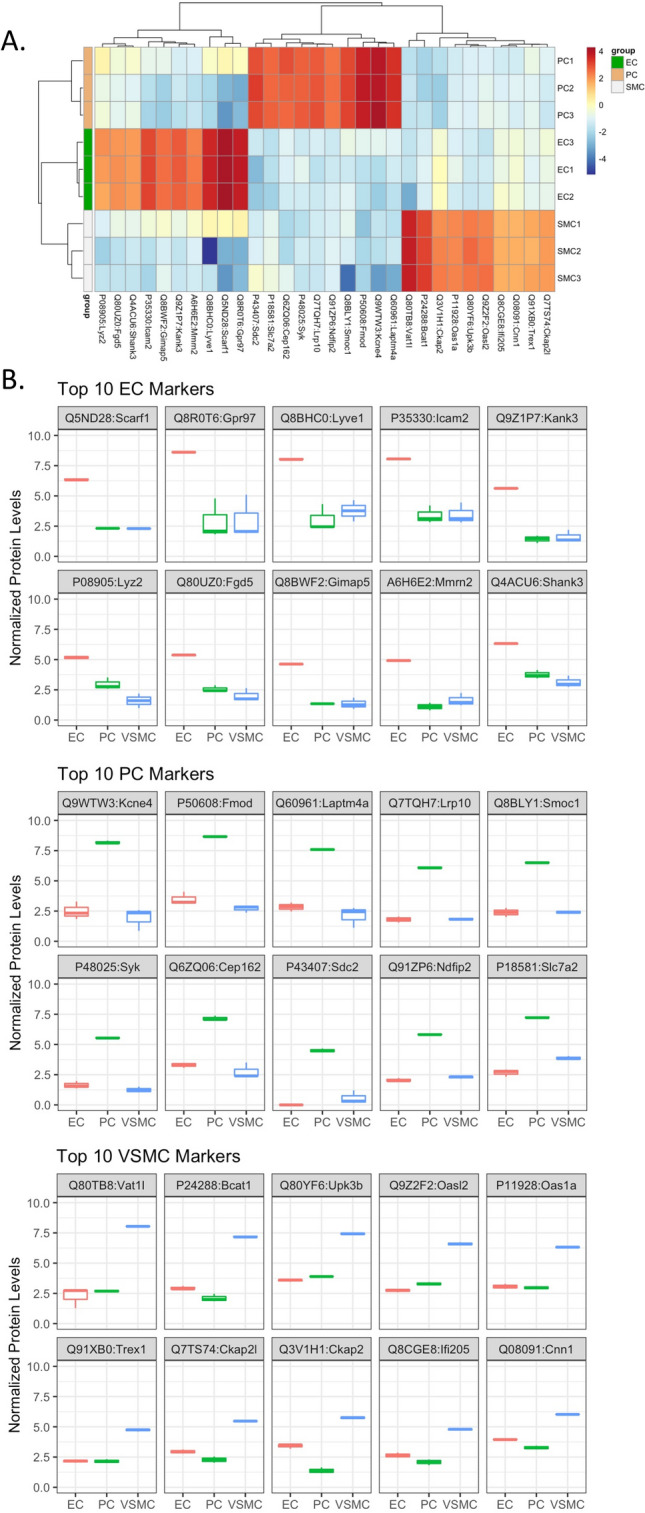
Top 10 novel differentially expressed protein markers in cultured ECs, PCs, and VSMCs. (**A**) Heatmap and (**B**) boxplots (median, interquartile range) of top 10 most abundant differentially expressed markers in each cell type, n = 3. Values were filtered based on FDR rate < 0.05 and then log fold change values compared to the other cell types. Additionally, data was filtered based on average cell type expression, with proteins with normalized log2cpm > 4 in cell type of interest and < 4 in the other 2 cell types.

**Figure 7:**
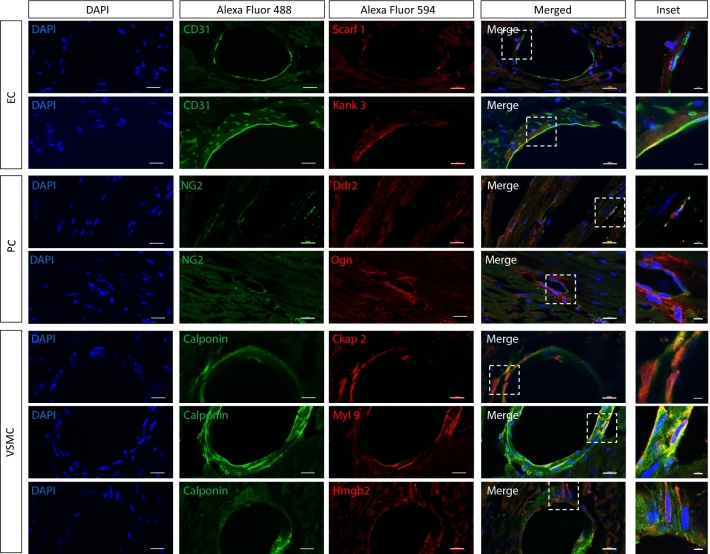
Validation of novel differentially expressed markers by immunohistochemistry of young adult mouse heart. IHC of 10-week-old male mouse heart, double labelling with novel differentially expressed markers identified by proteomic analysis of cell cultures (Figs. 6 and Supplemental Fig. S3). Novel markers (Alexa Fluor 594) are double-labelled with classical cell-specific markers (Alexa Fluor 488): CD31 for ECs, NG2 for PCs, calponin-1 for VSMCs; scale bar (DAPI, Alexa Fluor 488 and 594, merged), 20 µm, scale bar (inset), 5 µm.

We identified novel proteins marking each of the three cell types. Figure 6A is heatmap depiction of abundant proteins in the three cell types, demonstrating clear separation of among these cells. This heatmap was constructed using proteins that are present in one cell type and either absent or low in other cells based on a cutoff level of abundance. Using this approach, we have identified 30 proteins that clearly differentiate the three cell types and are also highly expressed in each cell type (Fig. 6A,B, and Table 1). For PCs, the following novel protein markers were identified: KCNE4, FMOD, LAP4A, LRP10, SMOC1, KSYK, CE162, SDC2, NFIP2, and CTR2. The following EC protein markers were found: SREC, AGRG3, LYVE1, ICAM2, KANK3, LYZ2, FGD5, GIMA5, MMRN2, and SHANK3. For VSMCs, the protein markers were: VAT1L, BCAT1, UPK3B, OASL2, OAS1A, TREX1, CKP2L, CKAP2, IFI5A, and CNN1. The boxplots in Fig. 6B compare levels of expression of these proteins among the three cell types. The full names, functions and genes encoding these proteins are summarized in Table 1 (see “Discussion” for additional information). Supplemental Fig. S3A is an alternative heatmap based on the largest fold difference relative to other cell types, which keeps all proteins detected in cells even if they are present at very low levels. Supplemental Fig. S3B are boxplots comparing levels of expression of these proteins among the three cell types. To validate some of these novel markers identified in Fig. 6 and Supplemental Fig. S3, we performed double labelling with classical cell-specific markers for cell type of interest by immunohistochemistry (IHC) (Fig. 7). Of the novel investigated, our IHC confirms expression of these markers specifically in the cell types identified by our proteomic analysis, either by co-localization (evident in yellow/ orange) with cell-specific marker in the case of Kank3/ CD31, Ddr2/ NG2, Ckap2/ calponin and Myl9/ calponin, or presence within the same cell as labelling for cell-specific marker in the case of Scarf1/ CD31, Ogn/ calponin and Hmgb2/ calponin, though with a different sub-cellular distribution to the classical cell-specific markers.

**Table 1:** Top 10 novel protein markers for ECs, PCs, and VSMCs.

Gene id	logCPM	PValue_ALL	FDR_ALL	PC vs. EC			VSMC vs. PC			VSMC vs EC			logCPM_EC	logCPM_PC	logCPM_VSMC	minFC
				logFC	Pvalue	FDR	logFC	Pvalue	FDR	logFC	Pvalue	FDR				
**PC**																
Q9WTW3:Kcne4	6.65	1.87E−50	5.61E−50	5.57	1.79E−27	5.92E−27	− 6.08	5.57E−31	1.72E−30	− 0.51	0.23	0.27	2.49	8.18	1.93	5.57
P50608:Fmod	7.15	6.75E−145	6.62E−144	5.11	3.80E−73	3.09E−72	− 5.93	1.22E−90	1.10E−89	− 0.82	6.03E−04	1.01E−03	3.48	8.67	2.71	5.11
Q60961:Laptm4a	6.09	8.94E−64	3.31E−63	4.72	5.80E−34	2.27E−33	− 5.33	1.51E−40	5.78E−40	− 0.61	0.07	0.09	2.84	7.59	2.10	4.72
Q7TQH7:Lrp10	4.66	4.4425745E−316	1.49066385E−314	4.28	5.45E−184	1.99E−182	− 4.26	1.95E−182	5.39E−181	0.02	0.86	0.88	1.80	6.10	1.84	4.26
Q8BLY1:Smoc1	5.06	3.23E−17	5.29E−17	4.09	3.61E−10	6.95E−10	− 4.61	5.49E−12	1.04E−11	− 0.52	0.37	0.42	2.39	6.51	1.35	4.09
P48025:Syk	4.13	5.18E−172	6.52E−171	3.89	3.50E−93	3.81E−92	− 4.29	8.02E−109	8.99E−108	− 0.40	0.02	0.03	1.64	5.55	1.25	3.89
Q6ZQ06:Cep162	5.75	2.69E−67	1.04E−66	3.87	4.71E−37	1.99E−36	− 4.30	1.13E−43	4.58E−43	− 0.43	0.11	0.14	3.29	7.16	2.77	3.87
P43407:Sdc2	3.05	1.71E−48	4.96E−48	5.16	4.52E−35	1.81E−34	− 3.86	7.35E−23	1.89E−22	1.30	2.71E−04	4.70E−04	− 0.78	4.49	0.56	3.86
Q91ZP6:Ndfip2	4.46	0.00E+00	0.00E+00	3.77	7.95E−225	4.25E−223	− 3.54	4.44E−203	1.40E−201	0.23	0.03	0.04	2.06	5.83	2.29	3.54
P18581:Slc7a2	5.85	9.61E−244	1.99E−242	4.55	2.07E−176	6.55E−175	− 3.37	1.10E−108	1.23E−107	1.19	3.77E−17	1.45E−16	2.67	7.24	3.87	3.37
**EC**																
Q5ND28:Scarf1	4.85	3.85E−09	5.17E−09	− 5.36	3.62E−06	5.74E−06	− 0.02	0.98	0.98	− 5.39	3.36E−06	6.92E−06	6.36	0.10	0.07	5.39
Q8R0T6:Gpr97	7.12	1.31E−09	1.79E−09	− 5.05	1.02E−06	1.68E−06	0.25	0.77	0.79	− 4.80	2.60E−06	5.42E−06	8.62	2.90	3.03	4.80
Q8BHC0:Lyve1	6.58	1.46E−08	1.93E−08	− 4.69	5.10E−06	7.98E−06	0.10	0.91	0.91	− 4.59	7.37E−06	1.47E−05	8.05	3.06	2.27	4.59
P35330:Icam2	6.58	1.98E−31	4.30E−31	− 4.52	1.09E−19	2.91E−19	0.13	0.76	0.78	− 4.39	7.14E−19	2.90E−18	8.04	3.39	3.46	4.39
Q9Z1P7:Kank3	4.20	4.80E−84	2.32E−83	− 4.17	3.69E−52	2.12E−51	0.21	0.38	0.41	− 3.96	4.01E−48	3.66E−47	5.62	1.42	1.59	3.96
P08905:Lyz2	3.98	8.50E−29	1.76E−28	− 2.15	1.20E−11	2.43E−11	− 1.34	1.42E−05	2.09E−05	− 3.50	2.05E−25	1.01E−24	5.17	2.96	1.59	3.50
Q80UZ0:Fgd5	4.10	4.63E−48	1.34E−47	− 2.78	2.43E−26	7.79E−26	− 0.50	0.04	0.05	− 3.28	1.42E−34	9.30E−34	5.36	2.57	2.01	3.28
Q8BWF2:Gimap5	3.26	1.96E−27	3.95E−27	− 4.10	1.66E−20	4.54E−20	0.88	0.03	0.03	− 3.22	2.94E−14	9.99E−14	4.62	0.32	1.35	3.22
A6H6E2:Mmrn2	3.56	5.39E−60	1.87E−59	− 3.77	7.72E−42	3.64E−41	0.58	0.02	0.03	− 3.19	4.82E−32	2.93E−31	4.90	1.11	1.64	3.19
Q4ACU6:Shank3	5.09	3.34E−46	9.36E−46	− 2.53	2.37E−24	7.18E−24	− 0.60	0.01	0.01	− 3.13	9.00E−35	5.93E−34	6.31	3.76	3.13	3.13
**VSMC**																
Q80TB8:Vat1l	6.51	2.39E−91	1.25E−90	0.28	0.34	0.37	5.35	2.62E−50	1.22E−49	5.63	3.93E−54	4.00E−53	2.26	2.68	8.03	5.35
P24288:Bcat1	5.72	3.08E−284	8.46E−283	− 0.82	3.28E−08	5.81E−08	5.06	1.20E−184	3.32E−183	4.24	8.03E−141	3.99E−139	2.94	2.10	7.18	4.24
Q80YF6:Upk3b	6.06	0.00E+00	0.00E+00	0.32	4.22E−05	6.30E−05	3.54	6.05547E−319	0.00E+00	3.86	9.88E−308	0.00E+00	3.57	3.89	7.43	3.54
Q9Z2F2:Oasl2	5.24	1.98239E−318	6.6969684E−317	0.55	6.68E−07	1.11E−06	3.31	3.00E−165	6.79E−164	3.86	3.21E−213	4.19E−211	2.74	3.29	6.60	3.31
P11928:Oas1a	5.00	1.19E−238	2.39E−237	− 0.10	0.39	0.42	3.34	4.45E−148	8.19E−147	3.24	1.38E−140	6.74E−139	3.07	2.97	6.32	3.24
Q91XB0:Trex1	3.57	6.63E−137	5.93E−136	4.06E−03	0.97	0.98	2.57	1.57E−86	1.32E−85	2.58	8.20E−87	1.66E−85	2.15	2.15	4.73	2.57
Q7TS74:Ckap2l	4.24	1.54E−126	1.21E−125	− 0.66	3.45E−06	5.47E−06	3.17	3.64E−94	3.34E−93	2.51	5.24E−63	6.64E−62	2.95	2.28	5.47	2.51
Q3V1H1:Ckap2	4.48	5.66E−174	7.34E−173	− 2.03	2.28E−41	1.06E−40	4.36	9.35E−152	1.82E−150	2.33	2.80E−53	2.81E−52	3.41	1.38	5.74	2.33
Q8CGE8:Ifi205	3.67	1.55E−80	7.13E−80	− 0.56	2.52E−04	3.61E−04	2.69	4.83E−62	2.78E−61	2.13	2.65E−41	2.05E−40	2.64	2.08	4.79	2.13
Q08091:Cnn1	4.91	8.67E−206	1.44E−204	− 0.68	1.15E−12	2.42E−12	2.74	1.16E−161	2.56E−160	2.06	2.63E−97	6.50E−96	3.96	3.28	6.02	2.06

## Discussion

We have developed a platform for isolating and culturing mouse heart primary microvascular cells, which we applied to define the proteomes of ECs, PCs, and VSMCs. We identified unique protein markers and a set of highly abundant proteins for each of the three cell types, and validated a subset by IHC of mouse heart tissue. These results will contribute to a better understanding of the physiological and pathophysiological mechanisms underlying coronary microvascular function and disease.

Recent advances in high-throughput sequencing and profiling technologies have accumulated a wealth of information on vascular cell transcriptomes^12^, proteomes^13^, and metabolomes^14,15^. The concept of “vasculome” has been introduced to integrate the various profiles of vascular cells and tissue. Unfortunately, the integration and interpretation of publicly available data remain a challenge, in part due to the differences in how and when data was collected. International standards have been introduced to allow the free and open exchange of large-format data^16^. However, more challenging than the variability in data processing and presentation is biological variability. A vast literature on vascular cell isolation from various organs describes multiple protocols for the isolation and culture of these cells^17–21^. Furthermore, gene expression in cells is highly dynamic, as it senses and responds to different environmental influences, changing over time and under physiological and pathophysiological states. To reduce the degree of biological variability, we developed a simple, reproducible workflow to isolate microvascular ECs, PCs, and VSMCs from mouse heart at the same time. Our approach reduces biological variability for two reasons: it focuses on microvascular cells, and it allows for simultaneous isolation of all three cell types from the same tissue. The microcirculation has unique properties, governed by unique expression of genes, proteins and metabolites that are distinct from those of large vessels. Despite these differences, many published studies did not distinguish between these two compartments. Furthermore, by concurrently isolating the three cell types, our approach ensures that all three isolated cells were exposed to same in vivo conditions. Finally, our approach is efficient and economical, as it saves time, animals, and reagents. In addition to standardizing cell isolation methods, our approach greatly improves co-culture studies investigating cell–cell interactions in the microcirculation^22,23^ by ensuring all cells used in the co-culture originated from the same tissue and were concurrently isolated using the same protocol.

We validated our protocol using three approaches: (1) heatmaps and PCA confirmed clear separation of proteomes from the three isolated cell types, (2) we used ICC to label isolated cells for known cell-specific markers, and (3) we confirmed the presence of these markers in vascular cell proteomes. Our proteomic analysis shows that levels of the canonical endothelial protein CD31 (PECAM1) was higher in ECs than in other cells, the canonical pericyte marker PDGFRβ higher in PCs than other cells, and the canonical α-SMA (ACTA2) and calponin (CNN1) markers higher in VSMCs compared to other cells. There were some differences between the markers detected by ICC vs. proteomics. For example, ICC labels ECs for vWF, but vWF does not show up among the top markers in EC proteome. This is not unexpected, given the differences in sensitivity between MS and ICC. Furthermore, vWF expression has been reported to be heterogeneous depending on the organ being studied^24^. On the other hand, while PDGFRβ was prominent in PC proteome, we were unable to find a good antibody to confirm this marker using ICC, highlighting the limitation of ICC reliance on antibody and immunolabeling techniques. For α-SMA, although it was clearly higher in VSMC proteome compared to other cells, ICC analysis showed positive labeling both in VSMCs and PCs. This is consistent with published reports showing expression of contractile proteins, including α-SMA, in PCs^25,26^. In contrast, NG2, which is expressed in both mural cells, was clearly positive in ICC, but did not show up as a top marker in PC proteome. Finally, 3G5 is not a protein; it is a ganglioside antigen that can be detected by an antibody^27^, but not MS-based proteomics.

Our study revealed novel markers for microvascular cells. Some, but not all markers have been reported to be expressed in vascular cells and play a role in physiological and pathophysiological processes in the microcirculation. For example, of the proteins differentially expressed in PCs in our study, KCNE4 (potassium voltage-gated channel subfamily E regulatory subunit (4) is expressed in vascular tissue, in addition to other smooth muscle tissues, including the uterus and the gastrointestinal tract^28^. Fibromodulin (FMOD) plays a role in angiogenesis during cutaneous wound healing^29^. Low-density lipoprotein receptor-related proteins (especially LRP10) clears amyloid β (Aβ) through VSMCs and plays a role in the pathogenesis of cerebral amyloid angiopathy (CAA)^30^. Secreted modular calcium-binding protein 1 (SMOC1) also plays a role in angiogenesis^31^. Syndecan-2 (SDC2) is expressed in the microvasculature of gliomas and regulates angiogenic processes in microvascular endothelial cells^32^. NDFIP2 is downregulated in the human umbilical vein endothelial cell line (HUVECs) under oxidative stress conditions^33^. Due to antibody availability, we were unable to immunohistochemically demonstrate expression of these proteins in heart tissue, in addition to our proteomic data, however we were able to validate PC-specific expression of two other novel marker identified by our proteomic data, Discoidin domain-containing receptor 2 (Ddr2) and Osteoglycin (Ogn), by IHC in the adult mouse heart.

Of the proteins differentially expressed in ECs, Scavenger receptor expressed by endothelial cells I (SCARF1/SREC-I) is expressed in endothelial cells and mediates the uptake of acetylated low-density lipoproteins^34^ and KANK3, a member of the KN motif and ankyrin repeat domain-containing protein family of adaptor proteins is involved in blood vessel development, although remains expressed in the adult^35,36^, both of which we validated as specifically expressed in ECs of the adult mouse heart by IHC. The adhesion molecule intercellular adhesion molecule 2 (ICAM-2) is expressed at the endothelial junctions and regulates angiogenesis^37^. FGD5, a Rho-family guanine nucleotide exchange factor, is a regulator of endothelial angiogenesis, apoptosis, and barrier function involved in stabilizing endothelial junctions^38,39^. Multimerin-2 (MMRN2), an endothelial extracellular matrix protein, is angiostatic by virtue of interfering with VEGF/VEGFR2 signaling, regulation of which occurs during tumor angiogenesis; it is also involved in cell–cell junctional stability^40–43^.

Among the proteins differentially expressed in VSMCs, a bioinformatic study identified synaptic vesicle membrane protein VAT-1 homolog-like (VAT1L) as being down-regulated in calcific aortic valve disease^44^. Cytosolic branched-chain aminotransferase (BCAT1) has been identified as a genomic biomarker for cardiotoxicity studies; it is also down-regulated in acute myocardial infarction and experimentally heart-specific upregulation of BCAT1 is protective against ischemic myocardial injury^45,46^. Mutations of Three-prime repair exonuclease 1 (TREX1) are found in retinal vasculopathy with cerebral leukoencephalopathy and systemic manifestations (RVCL-S), a small vessel disease, and are also associated with decreased cerebrovascular reactivity^47,48^. In mice, knock-out of TREX1 leads to inflammatory myocarditis leading to cardiomyopathy and circulatory failure^49^. Calponin-1 (CNN1) regulates VSMC contraction in physiology; however, it is abnormally expressed in pathological situations. It is in involved in the molecular changes underlying coarctation of the aorta, a serious innate heart disease, where it is highly expressed on the surface of the thickened intima^50^. Decreased expression of CNN1 is observed in the heart of dilated cardiomyopathy mouse models, with improved outcomes upon rescued expression^51^; it is also implicated in vascular calcification by VSMCs in vitro^52^. Of the novel proteins differentially expressed in VSMCs, we show cell-specific expression of High mobility group protein b2 (Hmbg2) in the adult mouse heart, which is implicated in hypertrophic growth and ischemia/reperfusion injury^53–55^, as well as Cytoskeleton-associated protein 2 (Ckap2) and Myosin regulatory light peptide 9 (Myl9); the functions of which are not yet known in the heart.

We identified differentially expressed proteins among the different cell types, as shown in the volcano plots. Monoamine oxidase A (MAOA) is expressed in endothelial cells^56^. Polymorphisms in the galactokinase (GALK1) gene is associated with peri-ventricular white mater lesion, a small vessel disease in brain^57^. Integrin alpha-V (ITGAV), transcript is down-regulated in human atherosclerotic arterial plaques^58^. Experimentally, renal expression of glutathione-S-transferase µ-type 1 (GSTM1) is reduced in hypertensive rats, with transgenic overexpression reducing hypertension in the stroke-prone spontaneously hypertensive (SHRSP) rat^59^. While in humans, loss of one or both copies *GSTM1* is associated hypertension in the elderly, as well as with kidney and heart failure^60,61^. Cytosolic non-specific dipeptidase (CNDP2) is differentially expressed between hypertensive and normotensive mice^62^. Disruption of Matrin-3 (MATR3) is associated with bicuspid aortic valve, aortic coarctation and patent ductus arteriosus in both mouse and human^63^. Cytosolic serine hydroxymethyltransferase (SHMT1) is implicated in calcification of VSMCs in hyperphosphatemia, such as in chronic kidney disease^64^. Finally, E3 ubiquitin-protein ligase RNF213 is a susceptibility allele for intracranial atherosclerosis, mutations of which are also associated with Moyamoa disease characterized by stenotic changes of the internal carotid arteries, peripheral pulmonary stenosis and pulmonary arterial hypertension^65,66^.

Our study has some limitations. Identifying cell-specific markers in the microcirculation is challenging because of the highly plastic nature of these cells with phenotypic switching under physiological and pathophysiological conditions following cell isolation and culture. As the examples presented above illustrate, some established markers reflect high level of expression, but that does not mean that these proteins are not expressed in other cell types. There is also discrepancy between markers detected at the level of the mRNA or protein, as demonstrated by VENN comparison of top protein and transcripts expressed in each cell type, showing 15–20% overlap. Finally, different methods of protein detection have different sensitivities, and in the case of ICC, detection is highly dependent on the antibody used and its titer. Another limitation is related to the mixed nature of cells isolated in our protocol. While our isolation protocol focuses on microvessels, it does not distinguish between arterioles, capillaries, and venules. For example, Lyve-1 (lymphatic vessel endothelial hyaluronan receptor-1) is predominantly associated with expression in the lymphatic endothelium; however, it does have a broader distribution; it has been reported in ECs of the lung, spleen, liver and the heart^67^, including the heart valve^68^, and has been suggested to be involved in uptake and degradation of waste products, including hyaluronan, from the blood and lymph^67^, especially during development^69^. The concern related to the mixed nature of our cell populations can be addressed using single-cell analysis methods, such as scRNAseq or single-cell mass cytometry (scCyTOF), which allows separation of cells from different vascular segment.

## Methods

To focus study on microvessels, the aorta and large vessels are removed from the surface of the heart before digestion. ECs are purified using EC-specific antibodies (CD31 and CD102) conjugated Dynabeads, PCs are isolated using magnetic Dynabeads linked to pericyte-specific 3G5 surface ganglioside^70–72^, and VSMCs are isolated after migration from heart slices incubated in the presence of VSMC-specific culture medium (Table 2). This study is reported in accordance with ARRIVE guidelines.

**Table 2:** Media composition.

Cell type	Component	Final concentration	Vendor	Catalogue number
Endothelial cells	DMEM		ThermoFisher Scientific	11,965–084
	+ Glucose	4.5 g/L		
	+ L-glutamine	4 mM		
	ECGS, BT-203	100 µg/mL	Biomedical Technologies Inc	J64516
	FBS	20%	Sigma-Aldrich	12103C
	Gentamicin	50 µg/mL	Sigma-Aldrich	G1397-10ML
	Heparin	100 µg/mL	Sigma-Aldrich	H3149-100KU
Pericytes	DMEM		Sigma-Aldrich	11,885–084
	+ Glucose	1 g/L		
	+ l-Glutamine	4 mM		
	+ Sodium pyruvate	1 mM		
	FBS	10%	Sigma-Aldrich	12103C
	Gentamicin	50 µg/mL	Sigma-Aldrich	G1397-10ML
Vascular Smooth Muscle Cells	DMEM		ThermoFisher Scientific	11,885–084
	+ Glucose	1 g/L		
	+ L-glutamine	4 mM		
	+ Sodium Pyruvate	1 mM		
	FBS	10%	Sigma-Aldrich	12103C
	Penicillin/ streptomycin	100 U/ml	ThermoFisher Scientific	15,140,122

### Preparation of 3G5 monoclonal antibody

Hybridoma cells, secreting 3G5 monclonal antibody, were purchased from ATCC (Bethesda, Maryland, #CRL-1814) and cultured according to ATCC guidelines. To produce 3G5 monoclonal antibody, the cells were cultured in serum-free DMEM for 10 days, supernatant collected, and antibody was isolated and purified with IgM Purification Kit (Thermo Fisher Scientific, Waltham MA, #44897). The subclass of antibody was identified and titered by mouse IgM ELISA kit (Life Diagnostics, West Chester PA, #5015-1) based on the manufacturer’s instructions.

### Preparation of 3G5-conjugated Dynabeads

Rat anti-mouse IgM Dynabeads (150 µL; Thermo Fisher Scientific, #11039D) were resuspended in 1 mL PBS + 0.1% bovine serum albumin (BSA) and mixed, the tube was placed on a magnetic separator (Dynal MPC-S) for 30 s and supernatant was removed. This step was repeated three times to ensure thorough washing of the beads. The beads were then resuspended in 3 × the original volume (450 µL) PBS + 0.1% BSA and 500 µL of purified 3G5 monoclonal antibody added (stock concentration 2.96 µg/mL). The tube was allowed to incubate overnight at 4 °C on a rotator set to low-medium speed. The beads were then washed again 3 × with PBS + 0.1% BSA to remove any unbound antibody from the solution. The beads were resuspended in 3 × the original volume PBS + 0.1% BSA, stored at 4 °C and used within 2 weeks.

### Preparation of CD31- and CD102-conjugated Dynabeads

Beads were prepared as above: sheep anti-rat IgG Dynabeads (Thermo Fisher Scientific, #11035) were conjugated to either rat anti-mouse CD31 (PECAM-1; BD Biosciences, Franklin Lakes NJ, #553369) or rat anti-mouse CD102 (ICAM-2) antibody (BD Biosciences, #553326). Five microliters of antibody (stock concentrations: 0.5 mg/mL CD102, 1.0 mg/mL CD31) was used to 150 µL Dynabeads.

### Isolation of mouse heart

For each preparation of cells, 5 10-week-old mice were used. Mice were sacrificed using isoflurane and cervical dislocation. Mice were placed supine, their chests soaked with 70% ethanol. An incision was made in the skin, and the rib cage was opened sagittally to expose the heart. The hearts were dissected out and placed into a 50 mL conical tube on ice containing 10 mL PBS, where they were pooled. Studies were performed according to the National Institutes of Health Guidelines for the use and care of laboratory animals, and protocols were approved by the OHSU Institutional Animal Care and Use Committee. Male mice were used here, however this protocol has also been used to successfully isolate and culture EC, PC and VSMC from female mice. A schematic overview of the isolation of each of the cell types is shown in Fig. 1. All subsequent steps were carried out in a laminar flow hood to ensure sterility.

### Isolation and culture of mouse heart VSMCs

The hearts were placed in a dish containing PBS, the aorta and large vessels were removed from the surface of the heart and discarded. The hearts were placed one-by-one into a coronal mouse heart slicer matrix, where 4 apical slices were made from each heart. The slices were placed into a collagen (Sigma-Aldrich, St. Louis MO, #C5533)-coated tissue culture plate, 1 slice per well of a 24-well plate, containing 100 µL FBS. The culture plate was placed into a cell culture incubator (37 °C; 95% air and 5% CO_2_) for 4 h, during which time the ECs and PCs were isolated from the remaining heart tissue (below). After 4 h of incubation, 500 µL of VSMC culture medium (see Table 2) was added to each well. The medium was changed 5 days later and incubated for a further 7 days, allowing for migration of VSMCs from the apical heart slices onto the tissue culture plastic. The slices were carefully removed from the wells using forceps (Dumont #5) and discarded. Cells were enzymatically detached from wells using trypsin: EDTA (0.05%: 0.5 M), once cells had detached 0.2 mL DMEM + 10% FBS per well was used to inactivate the enzyme. The cell suspension was transferred to a 15 mL conical tube, pooled, and centrifuged at 1000 rpm for 8 min. The supernatant was removed, cells were re-suspended in 10 mL of SMC culture medium and plated in collagen-coated plated/ dishes depending on experimental design.

### Isolation and culture of mouse heart ECs

The remaining heart tissue was placed in a dish containing PBS, the chambers were opened, and any coagulated blood was removed. The tissue was placed in a new dish and finely minced with a razor blade before being placed into a 50 mL tube containing 10 mL DMEM + 1 mg/mL collagenase (CLS-2; Worthington Biochemical Corporation, Lakewood NJ, #LS004176) + 1 mg/mL DNase I. Half-way through, and following, 45 min of gentle agitation at 37 °C, the tissue pieces were triturated using a 6″ long 14-gauge metal cannula attached to a 35 mL disposable syringe. Following the final trituration, the suspension was passed through a 70 µm disposable cell strainer and collected in a 50 mL conical tube, 10 mL DMEM was also passed through the cell strainer to collect any remaining cells. The cell suspension was centrifuged at 1000 rpm for 10 min at room temperature (RT). The cell pellet was resuspended in 6 mL DMEM + 60 µL CD31-conjugated Dynabeads in a 15 mL conical tube and placed on a rotator (medium setting) for 40 min at RT. The tube was mounted into a magnetic separator for 1 min allowing the beads (with cells attached) to adhere to the sides, the supernatant containing unbound cells was collected and used for pericyte culture (see below). The tube was removed from the magnet, the beads were resuspended in 3 mL DMEM and again placed into the magnetic separator. This was repeated 2–3 times until the supernatant was clear. The beads (with bound cells) were then resuspended in 10 mL endothelial culture medium (see Table 2) and plated in a collagen-coated T75 flask. Medium was replaced twice weekly, with cells approaching confluence at 8–10 days after plating.

Once confluent, cells were enzymatically detached using trypsin–EDTA (0.05%; ThermoFisher Scientific, #25300054); cells were rinsed 3X with PBS and incubated at 37 °C with 3 mL trypsin–EDTA until cells detached. Once detached DMEM + 10% FBS was used to inactivate the enzyme to a final volume of 10 mL. Cells were transferred to a 15 mL conical tube and centrifuged at 1000 rpm for 10 min at RT, following which medium was aspirated and cells were resuspended in 5 mL DMEM. Cells were placed onto the magnet for 1 min; medium containing unbound cells was collected and placed into a new 15 mL conical tube, the tube with bound CD31 beads was discarded. CD102-conjugated Dynabeads (50 µL) were added to the cells, the tube was placed on a rotator (medium setting) for 40 min at RT. The tube was mounted into a magnetic separator for 1 min allowing the beads (with cells attached) to adhere to the sides, the supernatant containing unbound cells was discarded. The CD102-bound cells were washed 3X as above and plated into 2 collagen-coated T75 flasks in 10 mL endothelial culture medium per flask, once again medium was replaced twice weekly, with cells approaching confluence at 8–10 days after plating.

### Isolation and culture of mouse heart PCs

The DMEM containing unbound cells collected from the CD31 sort of the endothelial culture (above) was plated in a collagen-coated T75 flask. The next day, once cells had adhered, the medium was replaced with 10 mL pericyte culture medium (see Table 2) and cells were grown until confluent, replacing the medium twice weekly. Once confluent, after approximately 7 days, the cells were rinsed × 3 with PBS and 2.5 mL DMEM containing 50 µL 3G5-conjugated Dynabeads was added. The plate was incubated at RT for 30 min with gentle agitation. Medium was replaced with 10 mL pericyte culture medium and incubated overnight. Cells were enzymatically detached using trypsin–EDTA (0.05%), as above, DMEM + 10% FBS was added to inactivate the enzyme and cells were centrifuged for 8 min at 1000 rpm. Supernatant was discarded, the cells were resuspended in 6 mL DMEM and placed into a magnetic separator for 1–2 min. The supernatant was removed, and the cells were twice washed with DMEM. The Dynabead-bound cells were then resuspended in 10 mL pericyte medium and plated in a collagen-coated T75 flask and grown until confluent (7–10 days), replacing medium twice weekly.

### Immunocytochemistry (ICC)

Cells, cultured on glass coverslips, were fixed in fresh 4% paraformaldehyde in PBS (0.1 M sodium phosphate buffer, 0.9% NaCl, pH 7.4) and subsequently blocked with 10% goat serum in PBS for 30 min, then incubated overnight at 4 °C with primary antibodies diluted in blocking buffer. The following primary antibodies and dilutions were used: mouse anti-alpha-SMA, 1:200 (Sigma-Aldrich, #A2547); rabbit anti-CD31, 1:100 (abcam, Cambridge UK, #ab28364); rabbit anti-PDGFR-β 1:100 (abcam, #ab32570); rabbit anti-vWF, 1:200 (Santa Cruz Biotechnology, Dallas TX, #SC-365712); rabbit anti-calponin-1 1:100 (Millipore, Burlington MA, #04-589); rabbit anti-NG2 chondroitin sulfate proteoglycan, 1:100 (Millipore, #AB5320); rat anti-CD90, 1:200 (abcam, #ab3105), and mouse anti-3G5 McAb, 1:200 (prepared as described above from hybridoma cells, ATCC, #CRL-1814, stock concentration 2.96 µg/mL). Cells were washed with PBS + 0.1% Tween 20 and secondary antibody (Alexa 568-conjugated donkey anti-rabbit or IgG-Alexa 546-conjugated donkey anti-mouse (Life Technologies, Carlsbad CA, #A10042 and #A10036)) was applied in blocking buffer for 2 h at RT. Cell nuclei were labeled with Hoechst 33342 (Life Technologies). The coverslips were washed and mounted using ProLong Gold antifade reagent (Life Technologies, # P36935). Images were acquired with a confocal microscope (Nikon Eclipse Tie-A1RSi).

### Immunohistochemistry (IHC)

Mouse myocardial left ventricular samples were fixed with fresh 4% paraformaldehyde. Tissues were cut by cryostat into 20-µm sections. The sections were mounted on superfrost glass slides. After 1 h of blocking in 10% NGS, sections were double labelled with following primary antibodies (rabbit anti-osteoglycin, Ogn, 1:50 (Proteintech, Rosemont, IL, #12755–1-AP); rabbit anti-discoidin domain-containing receptor 2, Ddr2, 1:50 (Novus Biologicals, Littleton, CO, #NBP-14926); rabbit anti-Scarf 1, 1:50 (ThermoFisher, #PA5-67923); rabbit anti- KN motif and ankyrin repeat domain-containing protein 3, Kank3, 1:50 (Novus Biologicals, #NBP2-82100); rabbit anti- cytoskeleton-associated protein 2, Ckap2, 1:50 (Mybiosource, San Diego, CA, #MBS2520053); rabbit anti- myosin regulatory light peptide 9, Myl9, 1:50 (Proteintech, #15354-1-AP); rabbit anti- high mobility group protein b2, Hmgb2, 1:50 (Proteintech, #14597-1-AP); rat anti-NG2 chondroitin sulfate proteoglycan, 1:50 (ThermoFisher, #MA5-24247); rat anti-CD31 (PECAM-1), 1:50 (BD Biosciences, # 550274); mouse anti-calponin, 1:50 (Santa Cruz, #sc-70487), followed by secondary antibodies: Alexa Fluor 488 donkey anti-mouse IgG, 1:200 (ThermoFisher, #A11029); Alexa Fluor 488 donkey anti-rat IgG, 1:200 (ThermoFisher, #A21208); Alexa Fluor 594 donkey anti-rabbit IgG, 1:200 (ThermoFisher, #A21207). All primary antibodies were diluted 10% NGS, and incubated overnight at 4 °C. All the secondary antibodies were diluted in PBS and incubated at RT for 2 h. After secondary incubation, sections were placed in an autofluorescence-reduction solution (10 mM CuSO4, 50 mM ammonium acetate) for 30 min, stained with DAPI (10 mg/ml, LifeTech, # H3570). The sections were viewed on confocal microscope, and digital images were taken with Nikon Imaging System (Nikon Eclipse Tie-A1RSi). Negative controls included sections in which the primary antibodies were omitted, incubated with secondary antibodies only, or sections in which both primary and secondary antibodies were omitted.

### Proteomics

#### Sample preparation for mass spectrometry

One T75 flask of cells was used per sample for proteomic analysis. Cells were lysed in 200 μl of 50 mM ammonium bicarbonate using a probe sonicator, centrifuged and protein concentration determined by BCA assay (Pierce, #23225). Thirty microgram of each sample was dissolved in 10 µl of 8 M urea, 1 M Tris, 8 mM CaCl_2_, 0.2 M methylamine (pH 8.5) solution, samples reduced by addition of 1 µl of 0.2 M dithiothreitol (DTT) and incubated at 50 °C for 15 min, followed by alkylation with 1 µl of 0.5 M idodoacetamide (IAA) and incubation at room temperate for 15 min. Excess IAA was removed by adding a further 2 µl of 0.2 M DTT, and proteins digested by diluting the urea concentration to 2 M and addition of 1.2 µg of sequencing garde grade trypsin (Promega). Following an overnight digestion at 37 °C, samples were acidified, and 20 µg of each digested sample was solid phase extracted using Oasis HLB 1 cc cartridges, dried by vacuum centrifugation, dissolved in 25 µl of 100 mM triethylammonium bicarbonate and labeled using TMT-10plex reagents (Thermo Scientific). The manufacturer-suggested protocol was used, except recommended amounts of each reagent were decreased by a factor of 4. Labeled samples were pooled and dried by vacuum centrifugation for mass spectrometric analysis.

#### Measurement of relative protein abundance by mass spectrometry

To increase numbers of quantified proteins, TMT labeled peptides were separated by two dimensions of reverse phase chromatography using a Dionex NCS-3500RS UltiMate RSLCnano UPLC system for sample loading and 2^nd^ dimension reverse phase separation, and a Dionex NCP-3200RS UltiMate RSLCnano UPLC system for dilution of the 1^st^ dimension reverse phase eluent. Twenty microliter samples containing 30 µg of pooled TMT-labeled digest were injected for 10 min onto a NanoEase 5 µm XBridge BEH130 C18 300 μm × 50 mm column (Waters) at 3 µl/min in a mobile phase containing 10 mM ammonium formate (pH 10), 2% acetonitrile (ACN). Peptides were then eluted by sequential injection of 20 µl volumes of 14, 20, 22, 24, 26, 28, 30, 40, and 90% ACN in 10 mM ammonium formate (pH 10) at 3 µl/min flow rate. Eluted peptides were then diluted at a tee with mobile phase containing 0.1% formic acid at a 12 µl/min flow rate. Peptides were delivered to an Acclaim PepMap 100 µm × 2 cm NanoViper C18, 5 µm trap on a switching valve. After 10 min of loading, the trap column was switched on-line to a PepMap RSLC C18, 2 µm, 75 µm × 25 cm EasySpray column (Thermo Scientific). Peptides were separated at low pH in the 2nd dimension using a 7.5–30% ACN gradient in mobile phase containing 0.1% formic acid at a 300 nl/min flow rate. Each 2nd dimension LC run required 2 h for separation and re-equilibration, therefore completion of LC/MS required 18 h. Tandem mass spectrometry data was collected using an Orbitrap Fusion Tribrid instrument configured with an EasySpray NanoSource (Thermo Scientific). Survey scans were performed in the Orbitrap mass analyzer (120,000 resolution), and data-dependent MS2 scans in the linear ion trap using collision-induced dissociation (NCE 35%) following isolation with the instrument’s quadrupole (2 Da isolation width). Reporter ion detection was performed in the Orbitrap mass analyzer (resolution 60,000) using MS3 scans following synchronous precursor isolation (top 10 ions) in the linear ion trap, and higher-energy collisional dissociation (NCE 65%) in the ion-routing multipole.

#### TMT data analysis

RAW instrument files were processed using Proteome Discoverer version 1.4.1.14 (Thermo Scientific) using SEQUEST HT software and a Swiss-Prot canonical mouse protein database (version 2014.05, 16,669 sequences). Searches were configured with static modifications for the TMT reagents (+ 229.163 peptide N-terminus and K residues) and alkylated cysteines (+ 57.021 C residues), variable oxidation of methionine (+ 15.995 M residues), parent ion tolerance of ± 10 ppm, fragment ion tolerance of 0.6 Da, monoisotopic masses, and trypsin cleavage (max 2 missed cleavages). Searches used a reversed sequence decoy strategy to control peptide false discovery^73^ and identifications were validated by Percolator software^74^. Only peptide-spectrum-matches with q-values ≤ 0.05 were accepted, and at least 2 distinct peptides had to be matched to a protein entry for its identification. Search results and TMT reporter ion intensities were exported as text files and processed with in-house Python scripts. A median reporter ion intensity cutoff of 1500 was used to reject low quality peptides, and all reporter ion intensities for unique peptides matched to each respective protein were summed to create total protein intensities. A minimum of 2 peptides contributing to the protein total was used to improve data quality. The mass spectrometry proteomics data have been deposited to the ProteomeXchange Consortium via the PRIDE^75^ partner repository with the dataset identifier PXD026673.

[The PRIDE data is available for reviewers: Username: reviewer_pdx026673@ebi.ac.uk, Password: xtEEv4MQ].

#### Differential protein expression analysis

Differential protein abundance was determined by comparing the total reporter ion intensities between groups using the R software (v 3.1.1) package edgeR (version 3.21.9;^76,77^), which performed data normalization, calculation of normalized log2 counts per million (log2cpm), multiple test correction, and calculation of false discovery rates (FDR). Test results are from likelihood ratio tests comparing each pair of cell types to each other, as well as a global likelihood ratio test that compares all three cell types. P-values less than 10^–350^ were truncated in Volcano plots.

To detect markers unique to each cell type, data was first filtered based on the FDR < 0.05 and then the log2 fold change (logFC) values, i.e., if a marker of PC was sought, first proteins with FDR comparing PC to VSMC < 0.05 and FDR comparing PC to EC < 0.05 were identified. Of those proteins, 10 were selected with the largest positive minimum log fold change comparing PC to EC and PC to VSMC. This analysis therefore identifies proteins that have the largest fold changes comparing the cell type of interest to the other two cell types. Additionally, data was filtered based on average cell type expression, selecting proteins with normalized log2cpm > 4 in the cell type of interest and < 4 in the other two cell types. The cutoff of 4 was selected as this was close to the 25^th^ percentile of log2cpm.

Clustered heatmaps of protein abundance of normalized log2cpm values were performed using Euclidean distance matrices. Principal Components Analysis (PCA) was performed on the normalized log2cpm values and the percent variance explained was calculated for the first two components. VENN Diagrams were calculated using number of differentially expressed proteins between cell type comparisons and drawn using BioVenn^78^. Gene Ontology (GO) analysis was carried out by the Search Tool for the Retrieval of Interacting Genes (STRING)^79^.

### RNA preparation and RNAseq analysis

RNA was extracted from confluent cultures of endothelial cells, pericytes and vascular smooth muscle cells (1 T75 flask/ sample) using TRIzol (Invitrogen) followed by RNAeasy universal mini RNA kit according to each manufacturer’s instructions. PolyA( +) RNA was next isolated using oligo-dT bound to magnetic beads and the recovered RNA was chemically fragmented to about 200–300 bp. Random hexamer priming was then used to produce double stranded cDNA and proprietary adaptors (Illumina) were attached to the fragments by ligation. The library is amplified by a limited number of rounds of polymerase chain reaction. The amplified library was cleaned using AMPure XP beads (Agencourt) and then run on the Bioanalyzer (Agilent) to ensure that successful library preparation. The HiSeq 2000 (Illumina) was used to sequence 100 cycle single reads and resulting data was converted to fastq files with CASAVA (Illumina). Subsequent trimming of known adapters and low-quality regions of reads was performed using Fastq-mcf and reads were assigned to genes using the program "featureCounts", part of the Subread R package. Reads that were aligned uniquely to reference sequence were used for further analyses; Tags with < 0.5 cpm in at least 2 samples were excluded. TMM normalization was performed with edgeR and log2cpm values were calculated.

## Supplementary Information

Supplementary Figure 1.

Supplementary Figure 2.

Supplementary Figure 3.

Supplementary Table 1.

Supplementary Table 2.

## Data Availability

The datasets presented here have been deposited into online repositories. The mass spectrometry proteomics data have been deposited to the ProteomeXchange Consortium via the PRIDE partner repository with the dataset identifier PXD026673. The RNA sequencing data have been deposited to GEO with the series number GSE190809.

## References

[1] Sinha A, Rahman H, Perera D (2021). Coronary microvascular disease: current concepts of pathophysiology, diagnosis and management. Cardiovasc. Endocrinol. Metab..

[2] Taqueti VR, Di Carli MF (2018). Coronary microvascular disease pathogenic mechanisms and therapeutic options: JACC state-of-the-art review. J. Am. Coll. Cardiol..

[3] Zetter BR (1981). The endothelial cells of large and small blood vessels. Diabetes.

[4] Aird WC (2012). Endothelial cell heterogeneity. Cold Spring Harb. Perspect. Med..

[5] Cleuren ACA (2019). The in vivo endothelial cell translatome is highly heterogeneous across vascular beds. Proc. Natl. Acad. Sci. USA.

[6] Vanlandewijck M (2018). A molecular atlas of cell types and zonation in the brain vasculature. Nature.

[7] Ross JM (2020). The expanding cell diversity of the brain vasculature. Front. Physiol..

[8] Bohannon, D.G., Long, D. & Kim, W.K. Understanding the heterogeneity of human pericyte subsets in blood-brain barrier homeostasis and neurological diseases. *Cells* **10** (2021). 10.3390/cells10040890 PMC8069782 33919664

[9] Lee J, Hyeon DY, Hwang D (2020). Single-cell multiomics: Technologies and data analysis methods. Exp. Mol. Med..

[10] Couvillion SP (2019). New mass spectrometry technologies contributing towards comprehensive and high throughput omics analyses of single cells. Analyst.

[11] Metz RP, Patterson JL, Wilson E (2012). Vascular smooth muscle cells: Isolation, culture, and characterization. Methods Mol. Biol..

[12] Chavkin NW, Hirschi KK (2020). Single cell analysis in vascular biology. Front Cardiovasc. Med..

[13] Lynch M, Barallobre-Barreiro J, Jahangiri M, Mayr M (2016). Vascular proteomics in metabolic and cardiovascular diseases. J. Intern. Med..

[14] Mayr M (2008). Proteomic and metabolomic analysis of smooth muscle cells derived from the arterial media and adventitial progenitors of apolipoprotein E-deficient mice. Circ. Res..

[15] Huang SF (2020). Cell-specific metabolomic responses to injury: Novel insights into blood-brain barrier modulation. Sci. Rep..

[16] Chervitz SA (2011). Data standards for Omics data: The basis of data sharing and reuse. Methods Mol. Biol..

[17] Gauthier SA, Sahoo S, Jung SS, Levy E (2012). Murine cerebrovascular cells as a cell culture model for cerebral amyloid angiopathy: Isolation of smooth muscle and endothelial cells from mouse brain. Methods Mol. Biol..

[18] Remillard, C.V., Makino, A. & Yuan, J.X.-J. Isolation and culture of pulmonary vascular smooth muscle and endothelial cells, in *Textbook of Pulmonary Vascular Disease*. (eds. J.X.J. Yuan *et al.*) 485–493 (Springer US, Boston, MA; 2011).

[19] Hewett PW, Murray JC (1993). Human lung microvessel endothelial cells: Isolation, culture, and characterization. Microvasc. Res..

[20] Siow RC, Pearson JD (2001). Vascular smooth muscle cells: Isolation, culture, and characterization. Methods Mol. Med..

[21] Crouch EE, Doetsch F (2018). FACS isolation of endothelial cells and pericytes from mouse brain microregions. Nat. Protoc..

[22] Alvino, V.V. *et al.* transplantation of allogeneic pericytes improves myocardial vascularization and reduces interstitial fibrosis in a swine model of reperfused acute myocardial infarction. *J. Am. Heart Assoc.* **7** (2018). 10.1161/JAHA.117.006727 PMC5850145 29358198

[23] Armulik A (2010). Pericytes regulate the blood-brain barrier. Nature.

[24] Pusztaszeri MP, Seelentag W, Bosman FT (2006). Immunohistochemical expression of endothelial markers CD31, CD34, von Willebrand factor, and Fli-1 in normal human tissues. J. Histochem. Cytochem..

[25] Bandopadhyay R (2001). Contractile proteins in pericytes at the blood-brain and blood-retinal barriers. J. Neurocytol..

[26] Attwell D, Mishra A, Hall CN, O'Farrell FM, Dalkara T (2016). What is a pericyte?. J. Cereb. Blood Flow Metab..

[27] Stramer BM (2004). Monoclonal antibody (3G5)-defined ganglioside: Cell surface marker of corneal keratocytes. Invest. Ophthalmol. Vis. Sci.

[28] Jepps, T.A., Carr, G., Lundegaard, P.R., Olesen, S.P. & Greenwood, I.A. Fundamental role for the KCNE4 ancillary subunit in Kv7.4 regulation of arterial tone. *J. Physiol.* **593**, 5325–5340 (2015). 10.1113/JP271286 PMC4704525 26503181

[29] Zheng Z (2014). Fibromodulin enhances angiogenesis during cutaneous wound healing. Plast. Reconstr. Surg. Glob. Open.

[30] Lane-Donovan C, Philips GT, Herz J (2014). More than cholesterol transporters: Lipoprotein receptors in CNS function and neurodegeneration. Neuron.

[31] Awwad K (2015). Role of secreted modular calcium-binding protein 1 (SMOC1) in transforming growth factor β signalling and angiogenesis. Cardiovasc. Res..

[32] Fears CY, Gladson CL, Woods A (2006). Syndecan-2 is expressed in the microvasculature of gliomas and regulates angiogenic processes in microvascular endothelial cells. J. Biol. Chem..

[33] Fukai T, Ushio-Fukai M, Kaplan JH (2018). Copper transporters and copper chaperones: Roles in cardiovascular physiology and disease. Am. J. Physiol. Cell. Physiol..

[34] Tamura Y (2004). Scavenger receptor expressed by endothelial cells I (SREC-I) mediates the uptake of acetylated low density lipoproteins by macrophages stimulated with lipopolysaccharide. J. Biol. Chem..

[35] Hensley MR (2016). Evolutionary and developmental analysis reveals KANK genes were co-opted for vertebrate vascular development. Sci. Rep..

[36] Guo SS, Seiwert A, Szeto IYY, Fässler R (2021). Tissue distribution and subcellular localization of the family of Kidney Ankyrin Repeat Domain (KANK) proteins. Exp Cell Res..

[37] Huang MT (2005). Endothelial intercellular adhesion molecule (ICAM)-2 regulates angiogenesis. Blood.

[38] Braun LJ (2020). Platelets docking to VWF prevent leaks during leukocyte extravasation by stimulating Tie-2. Blood.

[39] Nakhaei-Nejad M, Haddad G, Zhang QX, Murray AG (2012). Facio-genital dysplasia-5 regulates matrix adhesion and survival of human endothelial cells. Arterioscler. Thromb. Vasc. Biol..

[40] Zheng W (2021). Multi-omics analysis of tumor angiogenesis characteristics and potential epigenetic regulation mechanisms in renal clear cell carcinoma. Cell Commun Signal..

[41] Andreuzzi E (2017). The angiostatic molecule Multimerin 2 is processed by MMP-9 to allow sprouting angiogenesis. Matrix Biol..

[42] Pellicani R (2020). Multimerin-2 maintains vascular stability and permeability. Matrix Biol..

[43] Lorenzon E (2012). MULTIMERIN2 impairs tumor angiogenesis and growth by interfering with VEGF-A/VEGFR2 pathway. Oncogene.

[44] Zhang Y, Ma L (2019). Identification of key genes and pathways in calcific aortic valve disease by bioinformatics analysis. J. Thorac. Dis..

[45] Lai Q (2021). Oxoeicosanoid receptor inhibition alleviates acute myocardial infarction through activation of BCAT1. Basic Res. Cardiol..

[46] Nishimura Y (2013). Genomic biomarkers for cardiotoxicity in rats as a sensitive tool in preclinical studies. J. Appl. Toxicol..

[47] Hoogeveen ES (2021). Cerebrovascular reactivity in retinal vasculopathy with cerebral leukoencephalopathy and systemic manifestations. J Cereb. Blood Flow. Metab..

[48] de Boer, I. *et al.* spectral domain optical coherence tomography in retinal vasculopathy with cerebral leukoencephalopathy and systemic manifestations: A monogenic small vessel disease. *J Neuroophthalmol* (2021). 10.1097/WNO.0000000000001336 34334759

[49] Morita M (2004). Gene-targeted mice lacking the Trex1 (DNase III) 3'–>5' DNA exonuclease develop inflammatory myocarditis. Mol. Cell Biol..

[50] Liu A (2021). RNA sequencing analyses in infants patients with coarctation of the aorta. Hereditas.

[51] Lu D (2014). Calponin1 inhibits dilated cardiomyopathy development in mice through the εPKC pathway. Int. J. Cardiol..

[52] Furmanik M (2020). Reactive oxygen-forming nox5 links vascular smooth muscle cell phenotypic switching and extracellular vesicle-mediated vascular calcification. Circ. Res..

[53] Franklin, S. *et al.* Quantitative analysis of the chromatin proteome in disease reveals remodeling principles and identifies high mobility group protein B2 as a regulator of hypertrophic growth. *Mol. Cell Proteomics* **11**, M111.014258 (2012). 10.1074/mcp.M111.014258 PMC3433888 22270000

[54] Li Y (2021). microRNA-130a-5p suppresses myocardial ischemia reperfusion injury by downregulating the HMGB2/NF-κB axis. BMC Cardiovasc. Disord..

[55] Boureima Oumarou D (2019). Involvement of microRNA-23b-5p in the promotion of cardiac hypertrophy and dysfunction via the HMGB2 signaling pathway. Biomed. Pharmacother..

[56] Burridge KA, Friedman MH (2010). Environment and vascular bed origin influence differences in endothelial transcriptional profiles of coronary and iliac arteries. Am. J. Physiol. Heart Circ. Physiol..

[57] Armstrong NJ (2020). Common genetic variation indicates separate causes for periventricular and deep white matter hyperintensities. Stroke.

[58] Oksala N (2015). Kindlin 3 (FERMT3) is associated with unstable atherosclerotic plaques, anti-inflammatory type II macrophages and upregulation of beta-2 integrins in all major arterial beds. Atherosclerosis.

[59] Olson E (2019). Transgenic overexpression of glutathione S-transferase μ-type 1 reduces hypertension and oxidative stress in the stroke-prone spontaneously hypertensive rat. J. Hypertens..

[60] Tin A (2017). The loss of GSTM1 associates with kidney failure and heart failure. J. Am. Soc. Nephrol..

[61] Capoluongo E (2009). GSTM1-null polymorphism as possible risk marker for hypertension: Results from the aging and longevity study in the Sirente Geographic Area (ilSIRENTE study). Clin. Chim. Acta.

[62] Chiu CL (2014). Identification of genes with altered expression in male and female Schlager hypertensive mice. BMC Med. Genet..

[63] Quintero-Rivera F (2015). MATR3 disruption in human and mouse associated with bicuspid aortic valve, aortic coarctation and patent ductus arteriosus. Hum. Mol. Genet0..

[64] Boehme B (2018). Role of cytosolic serine hydroxymethyl transferase 1 (SHMT1) in phosphate-induced vascular smooth muscle cell calcification. Kidney Blood Press Res..

[65] Kim HJ (2021). Role of the RNF213 variant in vascular outcomes in patients with intracranial atherosclerosis. J. Am. Heart Assoc..

[66] Ruopp NF, Maron BA (2020). Pulmonary arterial hypertension in the modern era: The intersection of genotype and phenotype. J. Heart Lung Transplant.

[67] Zheng M, Kimura S, Nio-Kobayashi J, Iwanaga T (2016). The selective distribution of LYVE-1-expressing endothelial cells and reticular cells in the reticulo-endothelial system (RES). Biomed. Res. (Tokyo, Japan).

[68] Blancas AA, Balaoing LR, Acosta FM, Grande-Allen KJ (2016). Identifying behavioral phenotypes and heterogeneity in heart valve surface endothelium. Cells Tissues Organs.

[69] Gordon EJ, Gale NW, Harvey NL (2008). Expression of the hyaluronan receptor LYVE-1 is not restricted to the lymphatic vasculature; LYVE-1 is also expressed on embryonic blood vessels. Dev. Dyn..

[70] Nayak RC, Berman AB, George KL, Eisenbarth GS, King GL (1988). A monoclonal antibody (3G5)-defined ganglioside antigen is expressed on the cell surface of microvascular pericytes. J. Exp. Med..

[71] Sundberg C, Kowanetz M, Brown LF, Detmar M, Dvorak HF (2002). Stable expression of angiopoietin-1 and other markers by cultured pericytes: Phenotypic similarities to a subpopulation of cells in maturing vessels during later stages of angiogenesis in vivo. Lab Invest..

[72] Nees S (2012). Isolation, bulk cultivation, and characterization of coronary microvascular pericytes: The second most frequent myocardial cell type in vitro. Am. J. Physiol. Heart Circ. Physiol..

[73] Elias JE, Gygi SP (2007). Target-decoy search strategy for increased confidence in large-scale protein identifications by mass spectrometry. Nat. Methods.

[74] Kall L, Canterbury JD, Weston J, Noble WS, MacCoss MJ (2007). Semi-supervised learning for peptide identification from shotgun proteomics datasets. Nat. Methods.

[75] Perez-Riverol Y (2019). The PRIDE database and related tools and resources in 2019: Improving support for quantification data. Nucleic Acids Res..

[76] Robinson MD, McCarthy DJ, Smyth GK (2010). edgeR: a Bioconductor package for differential expression analysis of digital gene expression data. Bioinformatics (Oxford, England).

[77] McCarthy DJ, Chen Y, Smyth GK (2012). Differential expression analysis of multifactor RNA-Seq experiments with respect to biological variation. Nucleic Acids Res..

[78] Hulsen T, de Vlieg J, Alkema W (2008). BioVenn—A web application for the comparison and visualization of biological lists using area-proportional Venn diagrams. BMC Genomics.

[79] Szklarczyk D (2019). STRING v11: Protein–protein association networks with increased coverage, supporting functional discovery in genome-wide experimental datasets. Nucleic Acids Res..

