# Supplementary material for: Proteomic profiling of concurrently isolated primary microvascular endothelial cells, pericytes, and vascular smooth muscle cells from adult mouse heart
Source: Sci Rep. 2022 May 25;12:8835. doi: 10.1038/s41598-022-12749-6 (PMC9132906; doi:10.1038/s41598-022-12749-6)

Supplemental Figure S1. VENN diagrams illustrating overlap between top 50 proteins and transcripts. Listed are MGI gene names for overlap between protein and transcript for each cell type; those in bold appear in the top 50 of multiple cell types. Note: accession numbers are missing for some transcripts, resulting total RNA numbers < 50 (see Supplemental Table 1).

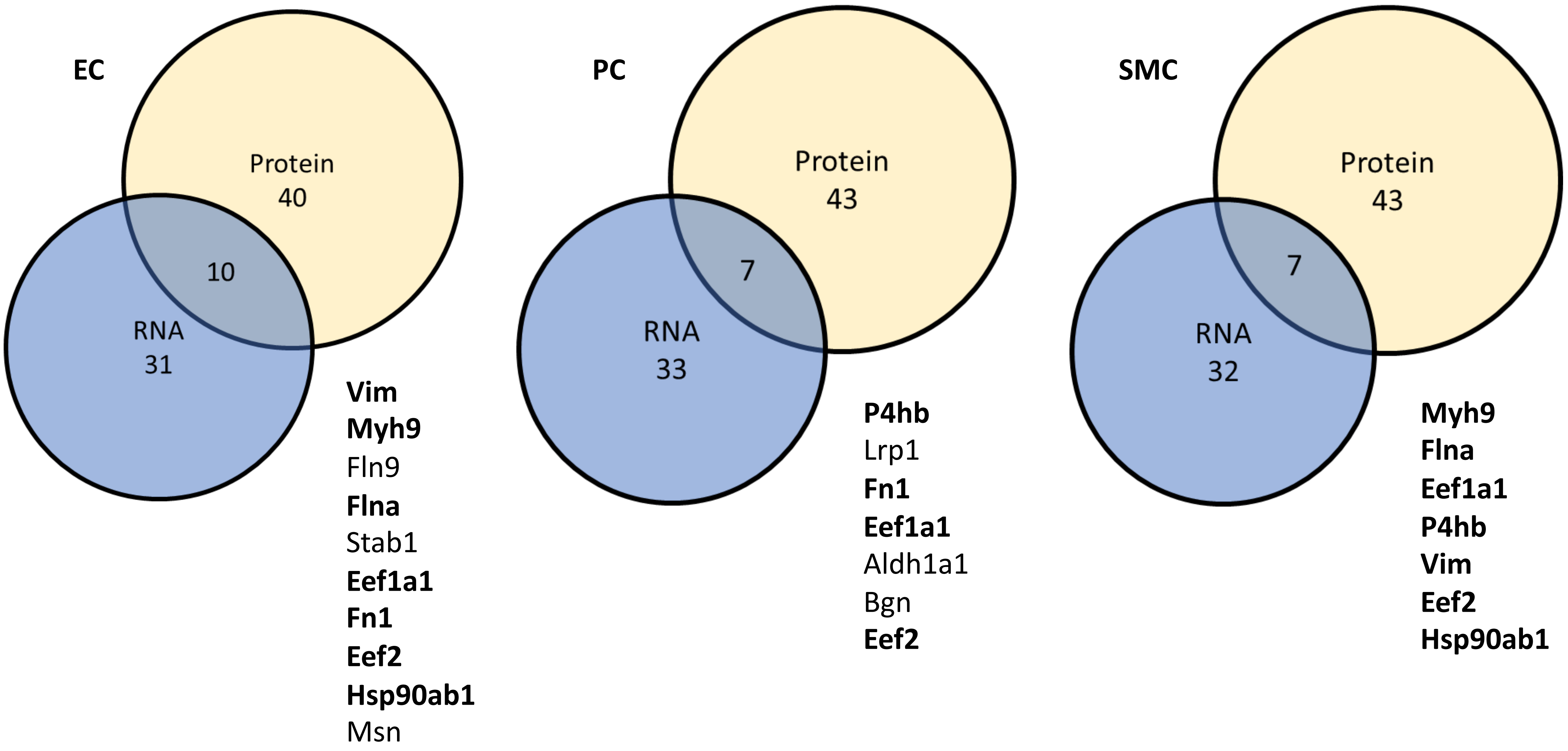

Supplement: Supplementary file 1 — Supplementary Figure 1. [file 41598_2022_12749_MOESM1_ESM.pdf]
