# Supplementary material for: Proteomic profiling of concurrently isolated primary microvascular endothelial cells, pericytes, and vascular smooth muscle cells from adult mouse heart
Source: Sci Rep. 2022 May 25;12:8835. doi: 10.1038/s41598-022-12749-6 (PMC9132906; doi:10.1038/s41598-022-12749-6)

Supplemental Figure S2. Gene Ontology (GO) term analysis for top 50 proteins (left) and transcripts (right) for ECs, PCs and VSMCs (listed in Supplemental Table 1). The vertical items are the name of the GO terms. The horizontal length represent count in network for each GO term. The colors denote the different GO categories.

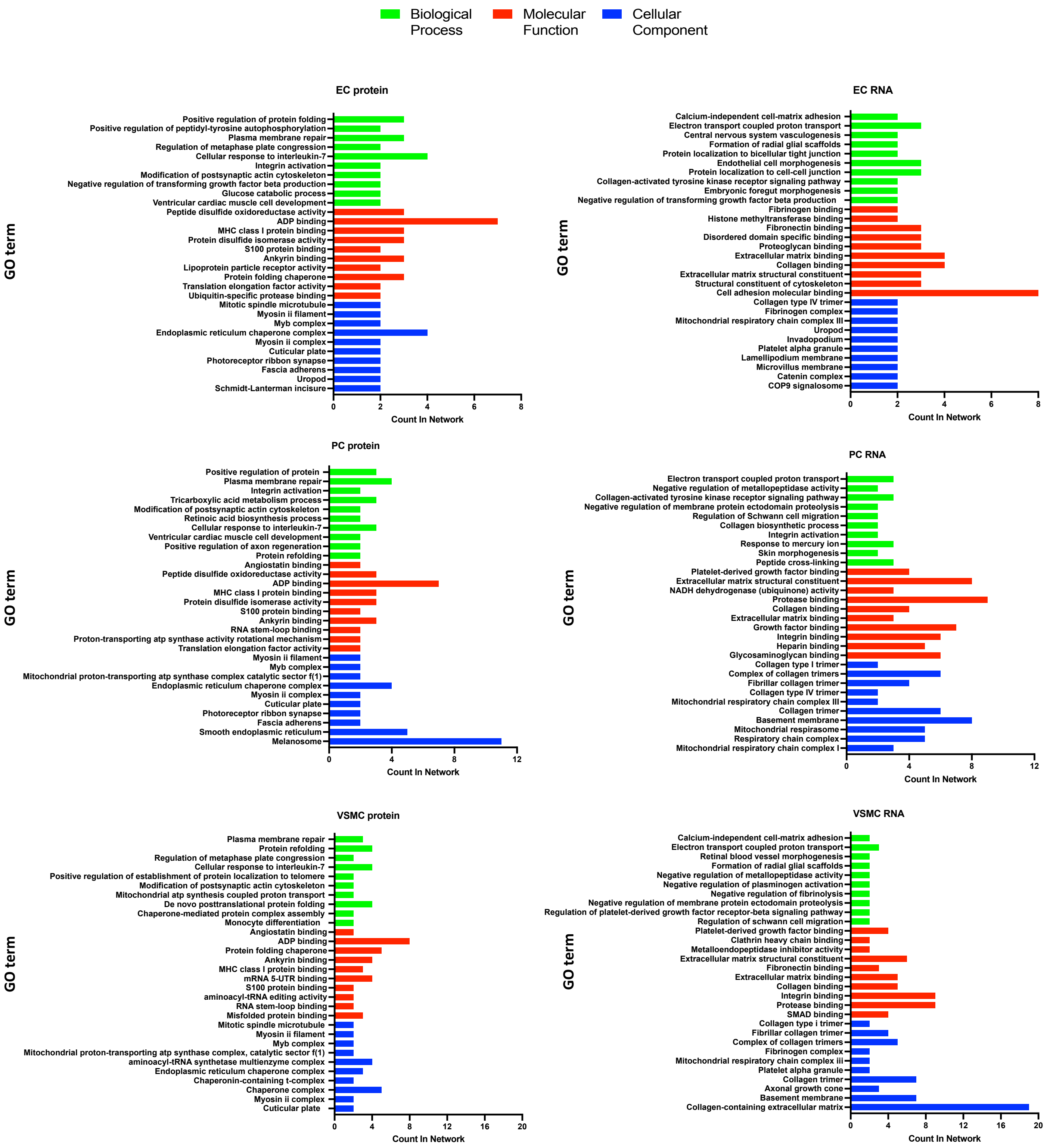

Supplement: Supplementary file 2 — Supplementary Figure 2. [file 41598_2022_12749_MOESM2_ESM.pdf]
