# Supplementary material for: Proteomic profiling of concurrently isolated primary microvascular endothelial cells, pericytes, and vascular smooth muscle cells from adult mouse heart
Source: Sci Rep. 2022 May 25;12:8835. doi: 10.1038/s41598-022-12749-6 (PMC9132906; doi:10.1038/s41598-022-12749-6)
Supplement: Supplementary file 3 — Supplementary Figure 3. [file 41598_2022_12749_MOESM3_ESM.pdf]

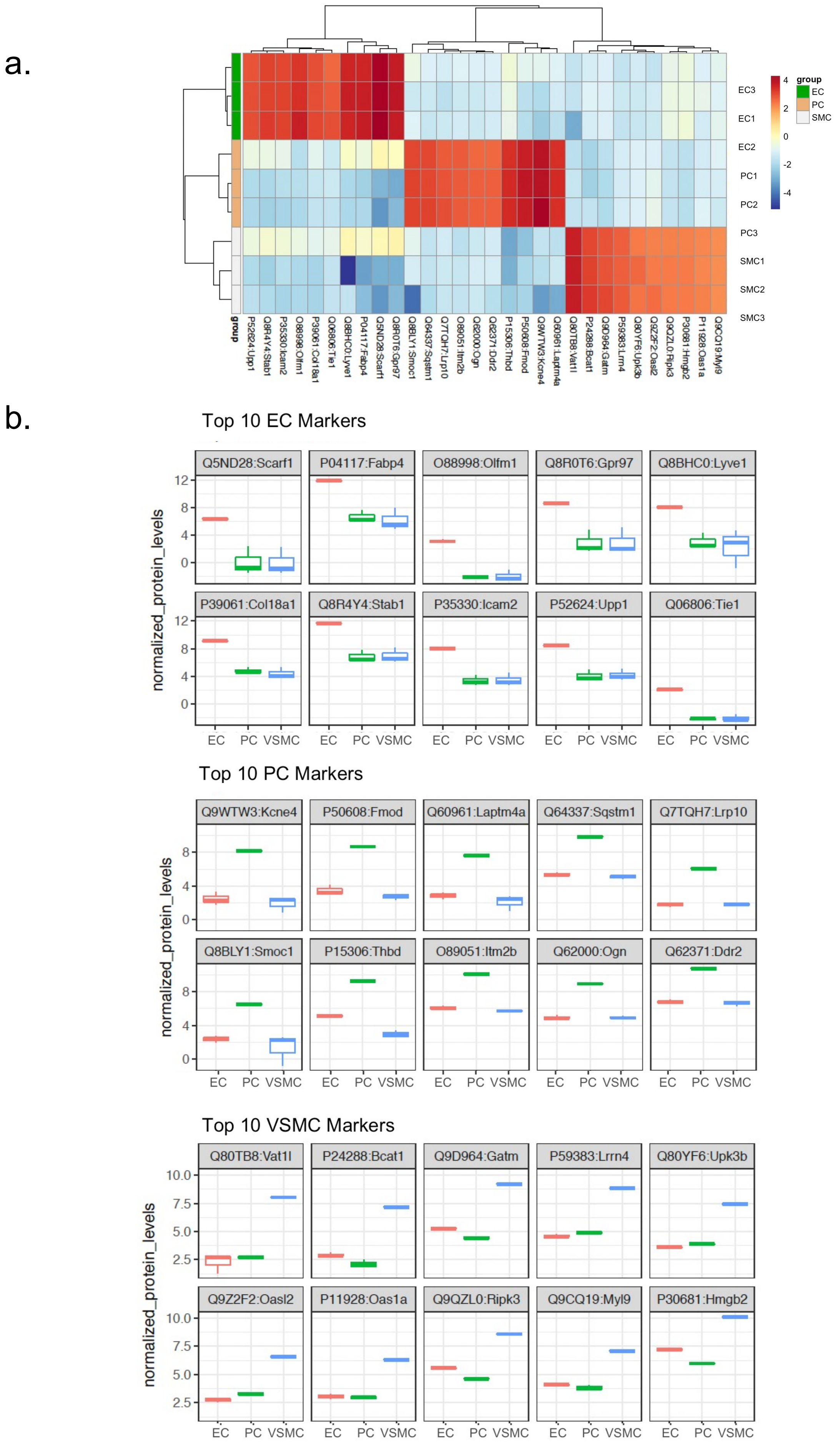

Supplemental Figure S3. Top 10 novel differentially expressed protein markers in cultured ECs, PCs, and VSMCs, based on fold change. A. Heatmap and B. Boxplots of top 10 most abundant differentially expressed markers in each cell type, n=3. Values were filtered based on FDR rate < 0.05 and then log fold change values compared to the other cell types.
